# Local Application of Scientific Research Practices Builds Student Engagement in Science and Environmental Health

**DOI:** 10.15695/jstem/v6i1.02

**Published:** 2023-02-15

**Authors:** David Jones, Anna Kiley, Michael Coe, Tony Ward, Carolyn Hester

**Affiliations:** 1 School of Public and Community Health Sciences, University of Montana, Missoula, MT; 2 Cedar Lake Research Group, Portland, OR

**Keywords:** Science, Student, Responses, Comments, Questions, Experiences, Skills, Learning, Interest, Survey, Air Quality, Real-world Scientific Research, Science Interest, Science Career Interest, Air Pollution

## Abstract

The University of Montana’s Research Education on Air and Cardiovascular Health (REACH) Program works with teachers to engage high school juniors and seniors in rudimentary real-world scientific research with the goals of improving their understanding of and interest in science, and to increase their interest in science careers. To evaluate the program, mixed-method approaches based on surveys that include both fixed-response and free-response questions for students has been used. Thematic analysis of student written responses to free-response questions provided evaluators with unanticipated student-centered information that was not targeted by the fixed-response questions. The analysis of student responses to free-response questions over a three to four-year period are the focus of this manuscript along with the implications such a STEM outreach program has to environmental and science education.

## INTRODUCTION

The Next Generation Science Standards (NGSS) emphasize that “science–and therefore science education–is central to the lives of all Americans” and indeed all citizens of all nations for “never before has our world been so complex and science knowledge so critical to making sense of it all” (NGSS, 2013). The NGSS are arranged around three dimensions: disciplinary core ideas (content), scientific and engineering practices, and cross-cutting concepts. This integration is a break from the traditional separation of the three into individual entities “leading to their separation in both instruction and assessment” (NGSS, 2013). Of the three dimensions, science and engineering practices have historically been neglected the most, either being taught separately or not at all (NGSS, 2013).

The mission of the University of Montana’s Research Education on Air and Cardiovascular Health (REACH) Program is to engage middle and high school students in rudimentary real-world scientific research to improve their understanding of and interest in science, and to increase their interest in careers in basic and clinical medical research. The program began in 2005 as the Air Toxics Under the Big Sky Program (2005–2012) and developed further into the Clean Air and Healthy Homes Program (2012–2018). Today the program is known as the REACH program, which focuses on indoor air pollution and cardiovascular health. The indoor pollutant that is most commonly measured as part of the REACH program is PM2.5. The EPA defines PM 2.5 as “fine inhalable particles with diameters that are generally 2.5 micrometers and smaller,” noting that PM 2.5 “particles come in many sizes and shapes and can be made up of hundreds of different chemicals” (United States Environmental Protection Agency, 2022). Not only does PM2.5 exposure result in adverse respiratory effects, but a growing body of research is showing the links between PM2.5 and cardiovascular health (Orr et al., 2020; Landguth et al., 2020). Globally, PM 2.5 exposure contributes to approximately 4 million deaths annually making it the fifth ranked risk factor for premature death (Thurston, 2018).

One of the most important aspects of the REACH program is its emphasis on local air quality research as a low-cost way to engage students in science practices – doing science. Students learn about timely and important environmental and health science content in parallel with learning science practices. Because the focus is on local air quality, the REACH program can be applied worldwide in both rural and urban settings and is easily tailored to addressing local air quality issues. The program is also easily integrated into the local schools’ science or other curriculum. The relatively low-cost air sampling equipment needed is provided by the REACH program, and there are varying ways for teachers to implement the program within existing classes, so the entry costs are low for schools with limited resources. This adaptability makes it very easy to integrate the three NGSS dimensions within the REACH program, and within the classroom.

The REACH program and its predecessors have been described in previous manuscripts (Delaloye et al., 2016; Delaloye et al., 2018; Ward et al., 2016). There is a typical schedule of events for students who participate in the REACH program. At the start of the program, students learn background information about air pollution and impacts to health which often involves a member of the REACH team making an in-person or virtual presentation. Students are then organized into small two to four person groups and given guidance on conducting research. The student research groups then develop their own research projects investigating indoor air quality within their homes, schools, or other community settings to explore a variety of relevant and real-world science research questions related to indoor air quality and its impact on health. Via their teachers, students are provided with low-cost PM2.5 air sampling equipment and training so they can complete the data collection portion of their project. With guidance, students then process and analyze their data. Finally, towards the end of the school year, students practice science communication by presenting their research findings to others, including their peers. This takes place in a variety of ways, including local community events, culminating events such as the annual REACH symposia at the University of Montana campus, in class or school presentations to community members, at scientific conferences, or at other physical or virtual venues.

Research aimed at supporting the ongoing development of the program and evaluating the efficacy of early versions of the program has used mixed-method approaches based on surveys that include both fixed-response and free-response questions for students (Delaloye et al., 2016; Delaloye et al., 2018; Ward et al., 2016). Previous reports of quantitative findings from fixed-response questions provided evidence that the program may positively impact student self-efficacy for research skills as well as their performance on tests of science reasoning and experimental design; more than a third of students reported that their participation made them more interested in science as a content area and almost a quarter reported that participation increased their interest in a science career (Delaloye et al., 2018; Ward et al., 2016). “In addition, an overwhelming percentage of students (85%) rated their experience in the symposium component of the program as either ‘good’ (59%) or ‘excellent’ (26%) (Ward et al., 2016).

Unlike structured multiple choice or Likert-scaled questions, free-response questions can provide evaluators with additional contextual understanding of such quantitative findings along with unanticipated student-centered information that was not targeted by the fixed-response questions. Thematic analysis of student writing in response to free-response questions may result in the emergence of themes that provide additional insight into student perspectives and intended or unintended effects the program may have on participating students. Student responses to free-response questions over a three to four-year period are the focus of this manuscript.

## METHODOLOGY

Surveys were administered in person at the end-of-year symposium on the University of Montana campus. Data was collected at symposia between the years of 2014 and 2018. At these symposia, high school students (primarily juniors and seniors) presented their air quality research projects in either a poster or oral presentation format to their peers and a panel of judges. The surveys were administered on paper and was composed of approximately 35 items including both fixed-response and free-response questions. The hand-written responses were converted to text in an Excel spreadsheet. Table 1 lists four free-response questions that are the focus of this manuscript.

Using a process designed by the program’s external evaluator, a three-person panel of REACH personnel used thematic analysis (Braun and Clarke, 2006; Braun et al., 2019) to identify patterns across the responses. The panel read the responses to each question a total of three times. Initially, each reviewer read the recorded responses separately while independently identifying themes in the responses. The reviewers then convened and developed consensus on a framework for coding the main common themes in the responses to each question. During the second reading, again done separately, each reviewer assigned these thematic codes to statements in each response. A single statement could be coded with more than one theme if different parts of the comment could be assigned to different themes. Finally, the reviewers reconvened to compare their individual coding and resolve any discrepancies on how they had assigned codes.

## RESULTS

The first two survey questions analyzed were included in the 2014, 2015, 2016, and 2018 surveys. These questions asked the students to comment about what they learned from preparing their poster or presentation and what they learned from presenting their poster or presentation. A total of 426 individual student surveys were recorded. From those students a total of 337 comments about what was learned from preparing their poster or presentation were analyzed resulting in 425 classifications into eight themes. Another 318 comments about what was learned from presenting the research were analyzed resulting in 430 classifications into the same eight themes. Table 2 displays the themes that emerged from the student responses, along with example quotes from student comments and the percentage of student responses to these questions that were coded using each theme.

Each theme includes at least 3% of student responses that were categorized. Student comments about learning preparation and time management and learning effective speaking and presenting skills represented 28% and 17% of the categorized comments. Over one-fifth (21%) of student preparation responses and 5% of the presentation responses described learning about environmental health science research related to air quality or air pollution as a highlight of their participation in the program. More than half of student comments on presenting (52%) described gaining effective speaking and presenting skills as the most important lesson learned from giving their research presentation, while 17% highlighted this same lesson learned from preparation. Almost a quarter (23%) described the benefit of giving their presentation in general terms as a challenging, positive experience. Approximately 8% of the comments indicated the preparation was a challenging, positive experience.

The second group of free-response questions from the survey related to how the REACH program affected their interest in science and their interest in a science career. These questions were part of the 2015, 2016, and 2018 surveys. A total of 341 student surveys were recorded and analyzed, with 364 comments classified based on science interest and another 375 comments classified based on science career interest. Table 3 displays the eight themes that were identified to represent patterns in the student responses, as well as select student comments from the surveys.

Comments about how the program changed the students’ interest in science and interest in science careers indicated they valued the research experience (19% and 15.5%), valued the new knowledge or information they had acquired (16% and 8%), valued the application of science to solve a real-world problem (8% and 5%) and/or valued the new perspective about science (4% and 4%). Taken together these four themes represent 47% and 32% of the coded comments respectively.

Comments about how the program changed the student’s interest in science and interest in a science career indicated they were always interested in science (11% and 13%), were interested in science but not air quality science (13% and 20%), and/or they were not interested in science or a science career (10.4% and 14.7%). Taken together these three themes represent 34% and 48% of the comments respectively.

## DISCUSSION

Two major findings emerged from the student survey responses. First, many comments affirmed the value of the REACH program’s focus on providing a platform for students’ real-world science research experiences. Second, many comments pointed to unanticipated affects that fall outside the specific goals or focus of the REACH program. The in-depth review of student open-ended comments provided additional context for earlier quantitative findings suggesting that the program may improve student self-efficacy for research skills along with student performance on tests of science reasoning and experimental design and student interest in science and science careers (Delaloye et al., 2018; Ward et al., 2016).

In the realm of confirming the value for students of gaining real-world science experiences, their comments focused on gaining an appreciation for the science research experience, the role science plays in society, and the factors that influence air quality and related public health concerns. Students also commented about how science experiments are designed, conducted, and communicated. Additionally, student comments clearly indicate they valued learning about and experiencing the science communication aspect of the program. The opportunity to communicate research findings by presenting at a science symposium is one of the major components of the program and was highlighted in over 50% of the student comments. This echoed and expanded upon the earlier quantitative finding that 85% of students rated their experience in the symposium as either ‘good’ (59%) or ‘excellent’ (26%) (Ward et al., 2016).

One student’s comment, “It was interesting to be able to research a topic that applies to my life,” which was echoed by many, is particularly significant. This speaks to the relevance and contextualization the program brings to students’ science experience. With respect to relevance, Holbrook and Rannikmäe suggest that “science education should be regarded as ‘education through science’, rather than ‘science through education’” and that “the over-riding target for science teaching in school, as an aspect of relevant education, is seen in responsible citizenry, based on enhancing scientific and technological literacy” (Hofstein, 2011). Tovar-Gálvez points out that utilizing local environmental situations and problems helps to contextualize the curriculum and is a transferable teaching element (Tovar-Gálvez, 2021).

Regarding unanticipated effects, students reported gaining an appreciation for managing time successfully and navigating group-work dynamics. This speaks to the “21st century skills” value of the program, which had not been addressed by any pre-conceived fixed-response questions or statistical analyses. The term “21st-century skills” is generally used to refer to certain core competencies in modern work settings such as collaboration, digital literacy, critical thinking, and problem-solving—skills that advocates believe schools should teach to help students thrive in today’s world (Rich, 2010). These 21st century skills are difficult to teach directly, but rather are best woven into the fabric of the high school curriculum.

Another unanticipated finding is the rather large number of students (48%) who expressed in their free-response comments a pre-existing firm commitment to being interested in science or a science career or to being uninterested. This suggests that many have already formed opinions about their interest or career trajectory prior to arriving at high school or very early in their high school experience. Again, none of the fixed-response questions had anticipated or asked about this issue, but it emerged strongly from the thematic analysis and has important ramifications for program design and delivery.

These firm commitments confirm earlier research findings that student attitudes toward science and their self-concepts related to science are largely formed before high school. Especially for girls; middle school experiences (and courses taken) largely determine high school attitudes (Newel, 2015). Newel et al. (2015) point out that research “focused upon late elementary and middle school students, in particular, because student attitudes toward science and interest in science-related careers are established before students enter secondary school, possibly as early as age 11.” The ‘Age 14 Dip’ is referred to by Bennett et al. (2009) saying their “study provides ample evidence of the particularly sharp fall in positive attitudes (towards science) between age 11 and age 14, in keeping with the findings of the study by Galton et al. (2003).”

In addition to the patterns across student responses, individual student responses may provide some insight into the nuances of their reflection on their REACH experiences. For example, the following comments give some indication of how students formulated their responses:

“(The project) made me think about what makes our air quality bad and gave me an idea on how to fix it.”

“The program showed me how important it is to regulate different things for the health and safety of my community and make it fun to do.”

“The program increased my interest in science by seeing how much our environment contributes to health of a person.”

“Makes me think that science can resolve problems we have about our planet in the future.”

All four of these comments from different students share the recognition that there are environmental problems out there and science has a role in solving them. Another topic in the student responses is amplified in the following comments:

“I learned that gathering data isn’t easy and it takes a lot of hard work to pull off an experiment.”

“I learned that in all sciences conclusions lead to more questions.”

“Much time and thought needs to go into a research project and thinking about the global context of it all is important.”

“I learned how to portray my ideas to other people in a clean and organized way.”

All of these comments express a growing appreciation for the practices of science as a knowledge seeking and knowledge sharing endeavor.

There are a couple of limitations to this work. The data collected across the years 2014–2018 does not include data from the 2017 student cohort. This is because the free-response questions were not used in the 2017 version of the survey. Additionally, the questions that queried students’ science or science career interest was not used in the 2014 version of the survey. Another limitation of this work is the lack of comparison groups which would provide insight to the overall impact of the program.

## CONCLUSION

Student comments from evaluation surveys have been useful over the course of several years as a tool to inform the development and refinement of the REACH program by expanding the understanding gained through structured quantitative research approaches. The data revealed some anticipated and unanticipated results concerning the effects of students’ participation in the program. Student comments described gaining the kinds of experiences sought by educators focused on developing “21st century skills.” Over 10% of the students also expressed through their comments the strengthening of their existing interest levels in science and science careers.

Programs such as REACH can help students determine a trajectory beyond their high school years. Learning science by doing science gives students experience conducting research with little at stake, providing an opportunity for students to take stock and determine whether further investment of time and resources in science education is a good fit for them. Those who find the practice of science appealing are empowered to pursue it further. Those who are not inclined to concentrate further on science at least gain experiences in the practices of science along with a better understanding of how science works and its importance and contributions to modern society.

Another finding from this work further confirms that students often make decisions about whether or not science is a good fit for them before they are in high school. Positive science research experiences in the regular curriculum may be required at an earlier age in order to cultivate student interest and skills, with further science education experiences sustained and expanded upon at later ages.

The use of low-cost air quality sensors in classroom-based student-driven research projects is an effective way of integrating science practice and environmental education into the curriculum, building student understanding of the science process as well as their awareness of science’s role in understanding and solving our pressing environmental problems. Since air pollution is an applicable environmental concern in every community across the planet (Gardiner, 2021), these projects can be easily tailored to the local situations.

While these research experiences at the high school level clearly offer several benefits, it is not yet clear whether they have the effect of increasing students’ science interest or interest in science careers. Data reported here indicate that many students have relatively fixed opinions about science and science careers by early high school. Many student comments indicated that their pre-existing high interest in science and science careers was strengthened by their REACH experiences. Another thing that is not clear from these data is whether or not through participation in these research experiences students gain a deeper, more complete understanding of the nature of science and/or science and engineering practices, cross-cutting concepts, and core ideas. Although many students commented on learning science content and practices during their participation, future research that includes the use of comparison groups is needed to estimate the mean impact of the program across large groups of students.

The inclusion of engaging experiences with real-world science and engineering practices using a program that is flexible, supportive and has low barriers to entry can be transformative for some students. The following three student comments illustrate this:

“This program helped me to realize that there is a lot more to air quality than what meets the eye. Studies triggered my interest and allowed me to think more openly.”

“The program taught me I can work hard and have ability to conduct a thorough experiment and be confident in my skills.”

“The program taught me that I have the ability to accomplish anything I set my mind to and I became more interested in science.”

While none of these comments indicate a change in their individual trajectory towards the sciences or a science career, the experience appears to have stimulated intellectual growth and appreciation for science. Through science experiences, their horizons–the way they view the world–has been broadened.

## Figures and Tables

**Table 1.** Student survey free-response questions and years used in survey.

Survey Question^a^	Years Used
What were the most important things you learned as a result of preparing for your presentation or poster this past school year?	2014, 2015, 2016, and 2018
What were the most important things you learned as a result of giving your presentation or poster today?	
Please explain how the program did or did not change your interest in science.	2015, 2016, and 2018
Please explain how the program did or did not change your interest in a science career.	

a These questions were not used in the 2017 version of the survey.

**Table 2.** Themes in student comments about what they learned from preparing and presenting summaries of their air quality research projects.^b^

Theme	Description	Examples	Distribution of coded comments % (n)
1) Design and conducting an experiment.	Students stated that they learned how to design, conduct, and explain an experiment or science research project.	*How to clearly describe an experiment and its importance on the real world*. *It is important to have good data and to be able to accurately interpret this data*.	Preparing 11.0 (47) Presenting 3.0 (13)
2) Preparation and time management.	Students stated they learned that preparation and time management were instrumental in the success of their project.	*Time management and communication is extremely important over long periods of time*. *A project that spans over a year goes by faster than you think*.	Preparing 27.6 (117) Presenting 12.8 (55)
3) Working with a group.	Students stated that working with and coordinating with a group of fellow students was important to the success of their project.	*What it’s like to work with a group when it’s a challenge to get together*. *Working with your group collaboratively will help you succeed*.	Preparing 7.9 (34) Presenting 2.6 (11)
4) Air Quality knowledge and related information.	Students stated they gained new knowledge and appreciation of air quality and related environmental health science issues.	*Air quality is important and I did not realize how relevant it was*. *Particulate matter is and can be a problem for health*.	Preparing 21.7 (92) Presenting 5.2 (22)
5) Nature of science.	Students stated they learned something about the nature of science as an evolving process.	*You can’t assume science. Experimenting is key*. *I learned that in all sciences conclusions lead to more questions*.	Preparing 3.2 (14) Presenting 0.9 (4)
6) Challenging, positive experience.	Students stated that the project was a challenging yet positive and rewarding experience.	*Data gathering and sharing isn’t as easy as it seems*. *To be proud of the time and effort I put into my project*. *If you don’t try, you won’t learn*.	Preparing 8.3 (35) Presenting 22.8 (98)
7) New Skill: Effective speaking and construction of poster or presentation (science communication).	Students stated they learned a new skill that involved effective speaking about or presenting their project including how to relax/not stress.	*I learned that I could stand and be able to speak about something I had no knowledge on previously*. *How to portray my ideas to other people in a clean and organized way*.	Preparing 17.5 (74) Presenting 52.2 (225)
8) New Skill: Data management and/or analysis.	Students stated they learned a new skill that involved efficient and effective data management and analysis with the use of spreadsheets and graphing software. (Excel, Sheets)	*How to manage data/change data to graphs*. *We learned how to statistically analyze data*.	Preparing 2.8 (12) Presenting 0.4 (2)

b N = 425 student comment classifications about preparing their presentation and 430 comment classifications about giving their presentation.

**Table 3.** Themes in student comments about how the program affected their interest in science and science careers.^c^

Theme	Description	Examples	Distribution of coded comments % (n)
1) Valued the research experience.	Students’ comments indicated an appreciation of the research experience they gained in the program.	*It made my love of science great again because it let me have a hands-on experience in science*. *It made me think for myself rather than reading it out of a book*.	Science interest 19.0 (69) Science career interest 15.5 (58)
2) Valued the new knowledge and/or information.	Students’ comments indicated an appreciation of new knowledge and /or information they gained during the course of the program.	*The program made me realize how much PM2.5 can affect you, and all sorts of normal everyday things can harm you*… *made (me) want to learn more*.	Science interest 15.9 (58) Science career interest 7.7 (29)
3) Valued the application of science to solve problems.	Students’ comments indicated an appreciation of the application of science to solve problems or improve quality of life.	*It made me think about what makes our air quality bad and gave me an idea on how to fix it*. *It helped me to realize that no matter what field I base my career in I can make a difference*. *Being a part of something that will benefit future generations was a privilege*.	Science interest 7.7 (28) Science career interest 5.0 (19)
4) Valued the new perspective.	Students’ comments indicated an appreciation of the new perspective about science they had gained from the program.	*It changed my interest in science because it showed a lot more aspects that can be studied*. *It was really interesting and informed me on a lot of things I had never thought of before*.	Science interest 3.6 (13) Science career interest 4.3 (16)
5) Reinforced interest in science or science career.	Students’ comments indicated they had been interested in science or a science career prior to participating in the program.	*I already knew that I really liked science, wasn’t much to change on my opinion*. *I was already interested in science before, the project only solidified this thinking*.	Science interest 11.0 (40) Science career interest 13.6 (51)
6) Interested in science or science career, but not air quality.	Students’ comments indicated they had been interested in science or a science career prior to participating in the program, but they were not interested in air quality science.	*It did not greatly affect my outlook in science because it isn’t really related to the field that I am interested in*. *I did not really enjoy this project, so as long as the science I do in the future is on other topics that I like better, then I’ll be good*. *It expanded my knowledge on radon but I do not think I will be very interested in this field of science*.	Science interest 12.9 (47) Science career interest 19.7 (74)
7) Not interested in science or science career.	Students’ comments indicated they were not interested in science or a science career prior to or after participating in the program.	*I don’t like science at all so this program didn’t help or wouldn’t* *This program didn’t change my interest because science is never a subject I would like to study in the future*. *I have never really been interested in being a scientist*.	Science interest 10.4 (38) Science career interest 14.7 (55)
8) No reason given or other.	Students’ comments were not classifiable in one of the above themes or there was no comment provided.	*It was a project and I needed the grade to pass*. *Would have preferred to spend more time on other things, not the project*.	Science interest 19.5 (71) Science career interest 19.5 (73)

c N = 364 student comment classifications related to **science interest** and 375 comment classifications related to **science career interest.**

## ABBREVIATIONS

EPA: Environmental Protection Agency
NGSS: Next Generation Science Standards
REACH: Research Education on Air and Cardiovascular Health
